# Evaluating the duration of post-discontinuation therapeutic ampicillin exposures in preterm infants

**DOI:** 10.1038/s41372-026-02600-5

**Published:** 2026-03-13

**Authors:** Angelique E. Boutzoukas, Jennifer Le, Ryan Kilpatrick, Michael J. Smith, Matthew Laughon, Daniel Gonzalez, Kelly C. Wade, Rachel G. Greenberg, Melissa Babilonia-Rosa, Daniel K. Benjamin Jr, Michael Cohen-Wolkowiez, Kanecia O. Zimmerman

**Affiliations:** 1 Department of Pediatrics, Duke University School of Medicine, Durham, NC USA; 2 Duke Clinical Research Institute, Durham, NC USA; 3 Skaggs School of Pharmacy and Pharmaceutical Sciences, University of California, San Diego, La Jolla, CA USA; 4 Department of Pediatrics, Tufts University School of Medicine, Boston, MA USA; 5 University of North Carolina, Chappel Hill, NC USA; 6 Division of Medicine, Duke University School of Medicine, Durham, NC USA; 7 Department of Pediatrics, Children’s Hospital of Philadelphia, University of Pennsylvania Perelman School of Medicine, Philadelphia, PA USA; 8 Department of Pediatrics, University of Pennsylvania Perelman School of Medicine, Philadelphia, PA USA

**Keywords:** Therapeutics, Bacterial infection

## Abstract

**Objective:**

Determine if preterm infants have prolonged therapeutic post-discontinuation antibiotic exposures (PDAE) following empiric ampicillin.

**Study design:**

Prospective study of 27 infants ≤7 days and <37 weeks gestational age (GA) receiving ampicillin (200 mg/kg/day); 49 post-discontinuation PK samples were collected. Exposures were predicted using PK simulations and a prior ampicillin PK model. The probability of target attainment 24- and 36-h after the final ampicillin dose for various minimum inhibitory concentrations (MICs) and the duration of therapeutic PDAE was calculated.

**Result:**

At 24- and 36-h after ampicillin, the probability of exposures ≥1 μg/mL was 95% and 60%, respectively. PDAE (≥1 μg/mL) lasted a median 33 h (95% confidence interval: 15, 79), and varied with GA, from median 53 h (<28 weeks GA) to 27 h (34–36 weeks GA).

**Conclusion:**

Many preterm infants experience therapeutic exposures at least 24 h after the final ampicillin dose. Shorter courses could be considered.

## Introduction

Excess antibiotic use in preterm infants is common and associated with added morbidity and mortality. Preterm infants are commonly administered antibiotics, including ampicillin and gentamicin, after birth for empiric management of early onset sepsis (EOS). Despite an incidence of EOS estimated at <0.5% of all preterm births in the United States [1], 8% of all preterm infants go on to receive ≥5 days of courses of antibiotics [2]. These rates rise to 20 to 40% among very or extremely low birthweight infants. [3] Unnecessary antibiotic exposures are particularly worrisome in this population; in very low birthweight infants who received antibiotics but did not develop sepsis, longer antibiotic use in the first week of life was associated with double the odds of mortality. [4] In extremely low birthweight infants, each antibiotic day was shown to increase the odds of necrotizing enterocolitis and mortality. [5] In recent years, an emphasis on antimicrobial stewardship and risk stratification in the preterm infant population has resulted in declining trends of empiric ampicillin use [5] however, ampicillin remains the most commonly administered drug in the neonatal intensive care unit. [6] Therefore, stewardship efforts that minimize unnecessary antibiotic exposures in this population are critical. [7, 8]

One emerging strategy to promote antimicrobial stewardship in the NICU involves identifying the optimal antibiotic regimens that provide the necessary, but not excessive, exposure to cover the expected window of time to culture positivity. The traditional “48-h rule out” originates from early studies, including by Pichichero and Rowley, [9, 10] in which most neonatal pathogens were detected within 2 days using manual culture systems. Though these findings have long guided clinical practice, they predate the widespread adoption of automated blood culture technology. Contemporary studies assessing time to positivity (TTP) demonstrate that the vast majority of EOS-causing organisms are detected well before 48 h, often within 24-36 h. [11, 12] As diagnostic capabilities have improved, the window of time that is needed for empiric antibiotics to provide coverage has become more clearly defined.

While local TTP data should inform empiric regimens, recent data suggest that, due to maturational delays in drug clearance, preterm infants receiving ampicillin may continue to experience substantial antimicrobial exposure after the final dose. In 2018, the Food and Drug Administration (FDA) label for ampicillin was changed to include gestational age- and postnatal age-based dosing to account for maturational changes, based on a population PK study conducted by Tremoulet et al. [13] Using this model, Le et al. conducted simulations using a virtual cohort of preterm infants with gestational age (GA) <28 weeks, postnatal age (PNA) <7 days, and birthweight (BW) < 1500 g to estimate post-discontinuation antibiotic exposures (PDAE). [14] These simulations suggested that a standard 48-h course of a commonly used regimen of 200 mg/kg/day divided Q12H of ampicillin would be followed by therapeutic ampicillin concentrations sufficient to cover Gram-positive organisms (i.e., Group B Streptococcus and *Listeria monocytogenes*) for an average of 74 h (95% CI: 35–109 h) after the final dose. Additionally, the FDA-approved dosing of 50 mg/kg given every 12 h provided sufficient exposures for 48 h after only two doses.

Although PDAE may contribute meaningfully to the total duration of effective antimicrobial exposure, this phenomenon has not been clinically confirmed in preterm infants. We therefore aimed to confirm that preterm infants <28 weeks GA and <1500 g BW receiving ampicillin in routine care experience prolonged PDAE and evaluate whether PDAE similarly occurs in infants 28–36 weeks GA or ≥1500 g BW.

## Materials/subjects and methods

### Study design

We conducted a single-center, open-label, opportunistic pharmacokinetic (PK) study in preterm infants (<37 weeks GA) ≤7 days PNA receiving ampicillin per standard of care. Study enrollment dates were 11 November 2021 to 21 September 2022. Written informed consent for the study was obtained from the parents or legal guardians. The study was approved by Duke University Institutional Review Board (Pro000042519).

### Study procedures

Infants received ampicillin per standard of care, typically while awaiting results of evaluation for EOS shortly after birth. The decision to start ampicillin was not a part of the study, and no specific dosing was required; however, during the period of study, preterm infants at our center received ampicillin 200 mg/kg/day divided every 8 h, infused over 15 min. There was no minimum or maximum duration of ampicillin administration required by the study.

Whole blood samples (minimum 200 μL) were collected for ampicillin PK from enrolled infants within a preferred sampling window between 6 and 120 h after the final dose of ampicillin. Sample collection occurred at the time of standard of care lab draws and was collected primarily by venipuncture, with capillary heel-stick collection used when venous access was not available. Samples were processed identically regardless of source (see *Sample processing* section), and the assay used has been validated for the measurement of ampicillin in human plasma from various blood sources. Up to a maximum of 10 PK samples and 2 mL/kg total whole blood sampled per subject. We collected all samples in human EDTA plasma tubes and centrifuged the samples at 3500 × *g* for 10 min at 4 °C within 1 h of the blood draw. We then transferred the plasma into a second polypropylene tube and immediately froze specimens at −20 °C. Specimens were transferred to a −80 °C freezer typically within 8 h, but at maximum within 24 h.

Dosing information was recorded from the electronic health record (EHR), including documented start times, and PK sample collection times were recorded by study staff. We collected data from the EHR, including demographics (gestational age, sex, race, ethnicity, birthweight, and postnatal age and current weight closest to dosing and PK sample collection times). Race and ethnicity data were collected from EHR and are recognized as social constructs and not expected to be related to ampicillin clearance. For dosing events, PK sample collection times, and other events of interest, the closest demographic values, including weight and height, and laboratory values, including serum creatinine, plasma albumin, total and direct bilirubin, aspartate aminotransferase, and alanine aminotransferase, were recorded. When these values were not recorded at the same time as an event (dosing event, sample collection event, or simulated time), the nearest last observation was carried forward or the next observation carried backward within ±7 days.

### Sample processing

Plasma samples were sent to OpAns Laboratory (Durham, NC) for measurement of ampicillin concentrations. The longest time from sample storage at −80 °C to processing was 6 months, which is below the known stability of ampicillin in human EDTA plasma at 216 days at –80 °C. Sample analysis followed methods previously developed and validated by OpAns. [15, 16] Ampicillin was extracted from 10 μL of human plasma by protein precipitation using acetonitrile fortified with [2H5]-ampicillin as an internal standard. Calibration standards and quality control samples were prepared in drug-free K2EDTA human plasma. Samples underwent high-performance liquid chromatography with tandem mass spectrometric detection using a validated assay. The assay method used has a validated range of 0.05 to 50 μg/mL. Samples with concentrations above the quantitation limit (AQL) were evaluated for evidence of detector saturation (chromatographic peak broadening). If absent, AQL concentrations were reported as is. For concentrations below the quantitation limit (BQL), if the chromatographic peak response could be characterized (i.e., extrapolated peaks had reasonable signal-to-noise ratios), the extrapolated values were reported. Quality controls were nominal concentrations of ampicillin in human plasma at 0.06, 4.0, and 40 μg/mL, and intra-assay accuracy ranged from 99.2-111.2%. Accuracy greater than 100% signifies that the assay overestimated the amount of ampicillin in the sample relative to its nominal concentration; the range reported is within the FDA ± 15% deviation from nominal values, so it was considered acceptable.

### Concentration-time plots, comparison of observed and model-predicted values, and Monte Carlo simulations

**Fig. 1:**
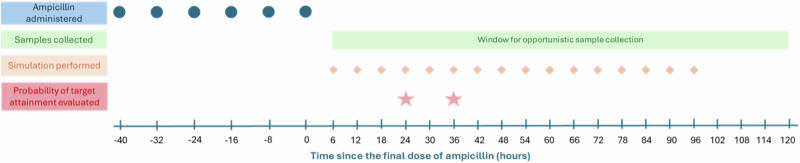
Example timeline of ampicillin administration, sample collection, and pharmacokinetic simulations. Example timing of ampicillin administration, opportunistic sample collection window, and timing of pharmacokinetic simulations performed for a typical enrolled infant. Post-discontinuation antibiotic exposure times are measured relative to the time of the final ampicillin dose. For each infant, in addition to the simulations performed every 6 h starting from the final dose of ampicillin through 96 h from the final dose, simulations were also performed to predict ampicillin concentrations at the time of any PK sample collection. The probability of target attainment analysis was conducted using the simulated values at 24 and 36 h after the final dose of ampicillin.

The observed ampicillin concentrations were plotted versus time since ampicillin discontinuation, or the time that the final dose of ampicillin was administered. Using the published ampicillin population PK model by Tremoulet et al., we conducted simulations with our prospectively-collected individual infant characteristics to predict antibiotic exposures after the final dose of ampicillin using NONMEM 7.3 (Icon, Dublin, Ireland). [13] The Tremoulet model was developed from a study of 73 infants (*n* = 142 ampicillin concentrations), including 21 infants born ≤34 weeks GA and ≤7 days postnatal age. [13] The model was a one-compartment model with linear elimination, and described that ampicillin clearance varies with weight, postmenstrual age, and serum creatinine. Here, we performed 500 simulations per infant to generate concentration-time profiles, inputting infant characteristics and the dosing events. Concentrations were predicted every 6 h starting from 6 h after the final dose of ampicillin (time 0) through 96 h after the final dose of ampicillin. Predicted concentrations were also simulated for each infant at the specific times that PK samples were collected. A timeline of ampicillin administration, PK sample collection, and PK simulations for a typical participant is shown in Fig. 1. The observed sample concentration was compared to the predicted concentrations (*n* = 500 simulations for each observed concentration) at that time point. With each simulation, NONMEM produces two predicted concentrations: the population predicted concentration, or PRED, based on fixed effects and the mean population parameter estimates, and the individual predicted concentration, or IPRED, predicted incorporating both fixed and random (individual-patient) specific random effects. The model-predicted concentrations were compared to the observed ampicillin concentrations by plotting population-predicted concentrations (PRED) and median individual predicted concentrations (IPRED) versus observed concentrations for within-range samples. For model comparison, AQL and BQL values were excluded to be consistent with the methods followed in developing the Tremoulet model. Notably, these individual predicted values were derived by sampling from the random effects distributions and incorporate unexplained interindividual variability, rather than model fitting of the newly collected observed data.

### Probability of target attainment analysis

The probability of target attainment (PTA) was the proportion of simulations (*n* = 27 infants, 500 simulations for each time point) where the individual predicted concentration (IPRED) was greater than various MIC targets of interest: 0.25, 1, 4, and 8 μg/mL. The PTA was calculated at 24 and 36 h after the final dose of ampicillin (see Fig. 1 for timeline). This analysis was conducted for the entire cohort as well as by GA subgroup: <28 weeks, 28 to <34 weeks, and 34 to 36 weeks.

For time-dependent antibiotics such as beta-lactams, the fraction of time spent above MIC (*f*T > MIC) is the pharmacodynamic endpoint that best relates exposures to efficacy, [17] with optimal bacterial killing occurring when concentrations are maintained above MIC for 40–50% of the dosing interval. We examined four MIC targets in the range of 0.25–8 μg/mL, which represent MIC susceptibility breakpoints for organisms commonly observed in the NICU. While lower MIC targets such as 0.25 and 1 μg/mL provide coverage for Group B *Streptococcus* and *Listeria monocytogenes*, respectively, higher MICs (4 and 8 μg/mL) are relevant for organisms such as *Enterococcus faecalis* and *E. coli*.

### Duration of post-discontinuation antibiotic exposures

To calculate the PDAE, or the number of hours following the final dose of ampicillin that the ampicillin concentration remained above a given MIC target, [14, 18] we used the formula $C={C_{0}}* {e}^{-{kt}}$, where C was the MIC target of interest, C_0_ was the minimum concentration at steady state (C_min,ss_) value simulated by NONMEM at the time of the final ampicillin dose, *k* was the elimination constant, derived from the simulations, and *t* was the PDAE time, in hours, at which the MIC target concentration was reached. We summarized PDAE in median and 95% confidence intervals (CI, calculated as the 2.5 and 97.5 percentile values for PDAE). We repeated this analysis for the various MIC targets and for each GA subgroup.

### Statistical analysis

Descriptive statistics were performed for demographic data and to summarize PDAE durations. Discrete-variable summaries included counts and proportions. For continuous variables, descriptive statistics included the number of observations, mean, median, and percentiles (2.5, 25, 75, 97.5). R Studio version 2022.07.1 was used for statistical analysis and graphical visualization of pharmacokinetic data.

## Results

The parents of 30 infants, 0–7 days PNA receiving ampicillin per standard of care, completed informed consent to participate in the study. Three enrolled infants had no PK samples collected. 100% of enrolled infants received ampicillin dosed 200 mg/kg/day divided into 3 daily doses, for a total of 6 doses. All enrolled infants received concomitant gentamicin. In the 27 infants who completed the study, 49 PK samples collected within 114 h following the final dose of ampicillin were included (Table 1). A comparison of the demographics of this enrolled study to those of the Tremoulet, et al. population PK model and the Le, et al., virtual simulation cohort is provided in Supplementary Table 1.

**Table 1:** Demographics of enrolled infants with at least one ampicillin concentration.

	Value^d^ (*N* = 27)
Birth weight (grams)	1730 [1310, 1970]
Min, Max	870, 2940
Actual weight^a^ (grams)	1650 [1310, 1970]
Gestational age	31w3d [29w6d, 33w2d]
Min, Max	26w6d, 36w6d
< 28 weeks	3 (11%)
28–34 weeks	18 (67%)
34–36 weeks	6 (22%)
Postnatal age^a^ (days)	0 [0, 1]
Sex, Female	16 (59%)
Race^b^	
Asian	1 (4%)
Black	13 (48%)
White	9 (33%)
Other	4 (15%)
Hispanic Ethnicity^b^	3 (11%)
Serum Creatinine (mg/dL)^a^	0.90 [0.80, 1.00]
Ampicillin daily dose (mg/kg/day)^c^	
200	27 (100%)
Number of Ampicillin doses received	6 [6, 6]

^a^At time of ampicillin administration.

^b^As reported in the electronic health record.

^c^Ampicillin daily dose administered divided every 8 h.

^d^All values are *n*, (%), or median [Q1, Q3], unless otherwise noted.

### Ampicillin concentrations over time and model performance

**Fig. 2:**
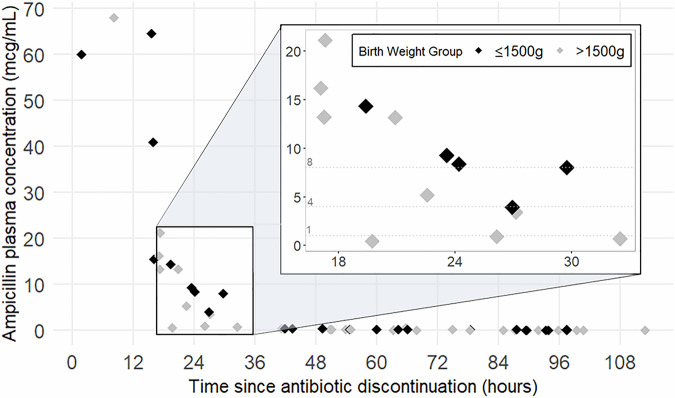
Ampicillin concentration time profile of post-discontinuation samples (*n* = 49). The graph displays the ampicillin concentration versus time profile for 49 plasma samples collected from 27 infants after the final dose of ampicillin that underwent high-performance liquid chromatography-mass spectrometry. All infants were ≤7 days postnatal age and received ampicillin starting on day of life 0 or 1, dosed at 200 mg/kg/day divided every 8 h for 6 total doses, to cover a 48-hour window of blood culture incubation. Time 0 represents the final ampicillin dose administered, approximately 40 h into therapy. One sample collected at 1.82 h after ampicillin discontinuation was collected prior to the planned sampling window for this study, but did not constitute a protocol deviation per the approved IRB.

Of 49 PK samples, three concentrations were above the upper quantitation limit (AQL) of 50 μg/mL, but there was no evidence of detector saturation for high concentration samples (chromatographic peak broadening), so concentrations were assumed to be reasonably accurate, and dilution and reanalysis were not pursued. One AQL sample was obtained 1.82h after ampicillin discontinuation, before the planned sampling window for this study; however, it did not constitute a protocol deviation per the approved IRB. Sixteen concentrations were below the quantitation limit (BQL). Because chromatographic peak responses were able to be characterized for all BQL samples, the reference laboratory reported these extrapolated values according to the standards outlined in Methods. The plasma concentration versus time profile following the final dose of ampicillin is shown in Fig. 2. Notably in the figure insert, between 18 and 30 h after the final dose of ampicillin, both infants <1500 g and ≥1500 g had ampicillin concentrations that remained above several relevant MIC breakpoints (0.25, 1, 4, 8 μg/mL).

**Fig. 3:**
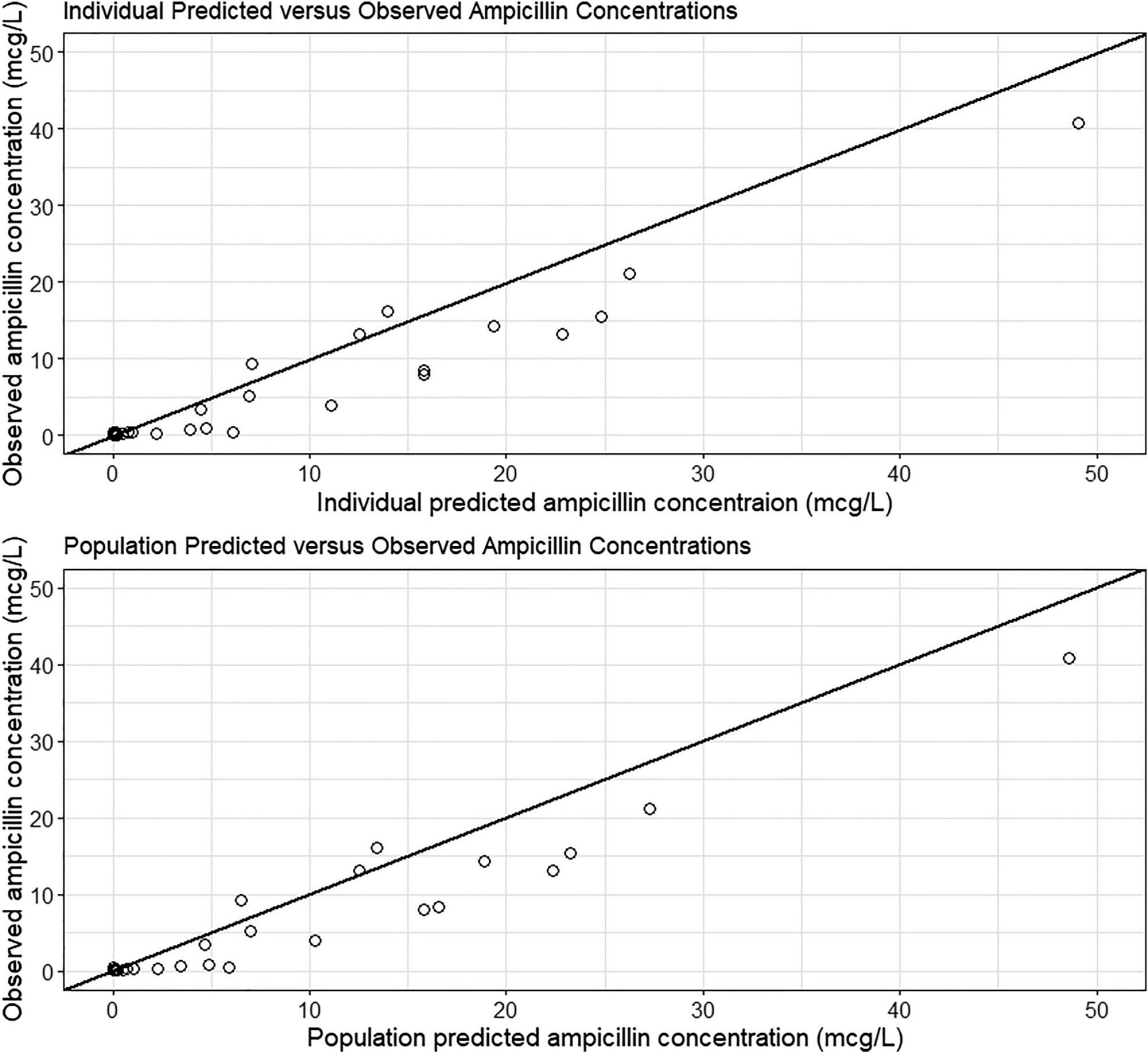
Predicted versus observed ampicillin concentrations. The graphs display predicted ampicillin concentrations versus observed ampicillin concentrations for *n* = 30 samples. Samples with concentrations outside of the validated range for the assay (0.05 to 50 μg/mL) were excluded from this figure, including 16 samples below the quantitation limit and 3 samples that were above the quantitation limit. The published Tremoulet model was used without refitting using the newly obtained data. For each infant, 500 simulations were conducted using individual infant characteristics to predict antibiotic exposures after the final dose of ampicillin using NONMEM 7.3 (Icon, Dublin, Ireland). The top graph shows individual predicted (IPRED) ampicillin concentrations on the horizontal axis, estimated using the characteristics from each of the infants at the time that each pharmacokinetic (PK) sample was collected; the median of these simulated IPRED values (*n* = 500 for each observation) was plotted. The bottom graph shows population predicted (PRED) ampicillin concentrations on the horizontal axis, calculated using fixed effects and estimated using the mean population parameters. The diagonal line is the line of unity, or a line indicating where predicted values and observed values are identical.

Model predicted concentrations (individual and population predicted values) were compared to the observed concentrations for within-range concentrations in Fig. 3. Overall, for within-range concentrations, the model predicted concentrations approximated the observed data points, though many observed concentrations fell slightly below the predicted values (Fig. 3). Because of their extrapolated nature, observed concentrations outside of the validated assay range (*n* = 19) are excluded from this figure evaluating model performance.

### Probability of target attainment analysis

**Fig. 4:**
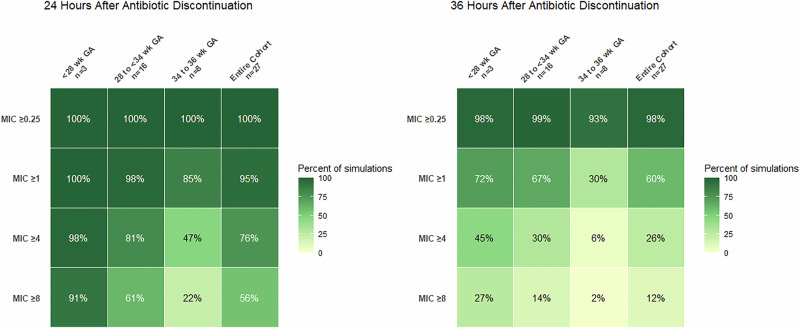
Probability of target attainment following the final dose of ampicillin. Each box of the heatmap represents the probability of target attainment (PTA) at a given minimum inhibitory concentration (MIC) target and for a given gestational age (GA) group. Each PTA estimate was calculated as the proportion of total simulations (500 simulations per infant) for which individual predicted concentration (IPRED) values were above a given MIC value. This was evaluated using simulated values 24- and 36-h after the final dose of ampicillin, shown in the left and right plots, respectively. The analysis was repeated for various MIC targets and by GA subgroups, as well as across the entire cohort of enrolled infants (*n* = 27).

The PTA at 24 and 36 h after the last dose of ampicillin provided ~40 h into therapy for various MIC breakpoints (0.25, 1, 4, 8 μg/mL) across the entire cohort and by GA group is shown in Fig. 4. As expected, the PTA was highest in the youngest gestational age groups. At 24 h after the final dose of ampicillin, the PTA was ≥95% across the entire cohort at an MIC target of 0.25 μg/mL and was >95% for all GA groups except those 34–36 weeks at an MIC target of 1 μg/mL. Even at higher MIC targets of 4 and 8 μg/mL, the most preterm infants (<28 weeks GA) retained a high likelihood of target attainment (>90%) 24 h after the final dose of ampicillin. Thirty-six hours after the final ampicillin dose, the PTA was >90% for all preterm infants at the low MIC target of 0.25 μg/mL; however, the PTA decreased with increasing GA. Subject-level plots of population predicted elimination curves and overlying observed concentrations versus time from 0 (time of final ampicillin dose) to 96 h are shown in supplementary Fig. S1, with close alignment of observed values to individual predicted curves.

### Duration of therapeutic post-discontinuation antibiotic exposure

Among all enrolled preterm infants, ampicillin administration was followed by a median PDAE of 33.2 h (95% CI 15, 79 h) at an MIC of 1 μg/mL (Table 2). The PDAE by GA group varied substantially, with a median of 53 h (95% CI 26–101) for infants < 28 weeks, while those 34–36weeks GA had a median PDAE duration of 27 h (95% CI 11, 54) at an MIC target of 1 μg/mL. Exposures remained above an MIC of 0.25 μg/mL, relevant for Group B Streptococcus, for a median of 35 to 67 h, depending on the GA group. At higher MICs (4 and 8 μg/mL), relevant for Gram-negative organisms such as *E. coli*, PDAE was shorter, but for preterm infants < 34 weeks, exposures remained above MIC targets (4 and 8 μg/mL) for a median of 22–40 h after the final dose of ampicillin.

**Table 2:** Duration of post-discontinuation antibiotic exposure (PDAE) above various minimum inhibitory concentration (MIC) values.

	Median (95% CI) PDAE^a^ (Time above MIC)^b^, in hours			
MIC (μg/mL)	≥ 0.25	≥ 1	≥ 4	≥ 8
Entire cohort (*n* = 27)	46.4 (20.8, 96.4)	33.2 (14.9, 78.6)	26.0 (9, 60.8)	20.9 (6, 51.9)
< 28 weeks GA (*n* = 3)	66.6 (33.4, 123)	53.2 (26.1, 101)	39.8 (18, 79.5)	33.1 (13.9, 68.6)
28–< 34 weeks GA (*n* = 18)	47.9 (23.4, 91.9)	37.5 (17.2, 74.7)	27.1 (10.9, 57.6)	21.9 (7.81, 49.1)
34 - 36 weeks GA (*n* = 6)	35.1 (16.9, 67.7)	26.6 (11.4, 53.7)	18 (6.29, 39.6)	13.8 (3.69, 32.9)

*CI* Confidence interval, *GA* gestational age, *MIC* minimum inhibitory concentration, *PDAE* post-discontinuation antibiotic exposure

^a^PDAE was calculated using the simulated minimum concentration at the time of the final dose of ampicillin, assuming steady state conditions (C_min_,_ss_) and solving for the time that the concentration would reach a target MIC level, using C=C_0_*e^-kt^. For each enrolled infant, 500 simulations were conducted; therefore, the number of total simulations contributing to this analysis is n*500 for each subgroup.

^b^Time from the final dose of ampicillin.

## Discussion

This prospective, opportunistic study is the first to document post-discontinuation therapeutic antibiotic exposures in preterm infants receiving empiric ampicillin. PDAE increases with decreasing gestational age, and depending on the dose administered and the target MIC, can result in prolonged and unrecognized antibiotic exposures. The target MIC to be considered therapeutic for ampicillin is a potential area of discourse among neonatologists. *E. coli* isolates in preterm infants have substantial rates of ampicillin resistance (66–83%) [1, 19], and American Academy of Pediatrics guidelines state that gentamicin provides empiric coverage for *E. coli* for preterm infants. [20] Therefore, we consider an MIC of 1 μg/mL, sufficient for optimal bacterial killing for Group B *Streptococcus* and *Listeria monocytogenes*, to be the relevant target during EOS evaluation in which gentamicin is also administered. Here, we demonstrated that ampicillin exposures exceed this target for more than 24 h (median 33.2 h, Q1 14.9, Q3 78.6) after the final dose of ampicillin at a dosage of 200 mg/kg/day divided every 8 h for 6 doses in 95% of simulated infants. These findings suggest that commonly used empiric dosing regimens—higher than FDA recommendations—may provide excess exposure beyond the diagnostic window, highlighting an opportunity to refine empiric therapy and advance neonatal antimicrobial stewardship.

Our findings add to a growing body of evidence suggesting that preterm infants may safely receive shorter courses of empiric ampicillin. The traditional 48-h duration for EOS evaluations originated with manual blood culture systems but persists despite modern automated TTP data showing that most EOS pathogens are detected well before 48 h. In a large multicenter cohort, Kuzniewicz et al. reported that 68% of positive EOS cultures resulted in 24 h, 94% in 36 h, and 97% in 48 h. [11] Coupled with accumulating evidence linking unnecessary antibiotic exposure in preterm infants to adverse outcomes [4, 5, 21, 22], these data have prompted NICUs to reconsider the duration of empiric EOS evaluations. Guided by local TTP data, Kumar et al. and Sanchez et al. implemented 24-h evaluations for EOS with ampicillin 100 mg/kg 12H. [23, 24] Across 6 NICUs, Sanchez et al reported reduced antibiotic re-initiation, no difference in positive cultures following antibiotic discontinuation, and no mortality difference. [24] Similarly, Kumar et al. reported no adverse safety signal following shortened empiric courses. [23] While no safety signal was identified in these studies, there were a few infants with positive cultures—0.7% (3/414) in Sanchez et al, and 2.8% (8/278) in Kumar et al—and larger studies powered to detect important safety endpoints are needed to confirm the findings. [23] Our data support these efforts by demonstrating that, at commonly used daily dosing of ampicillin, preterm infants experience sustained post-discontinuation exposures extending beyond the time to culture positivity. Although empiric antibiotics were stopped after 48 h in our cohort, prior simulations by Le et al. in extremely preterm infants (22–25 weeks GA) predicted that even 24-h courses at similar daily doses would be followed by prolonged therapeutic concentrations. [18]

Physiologic immaturity of renal elimination pathways helps explain the prolonged PDAE and changes across GA groups observed. Ampicillin is cleared through both glomerular filtration and active tubular secretion, and both pathways undergo substantial ontogenic maturation. [25] In the most premature infants, limited nephrogenesis and reduced glomerular filtration rate and transporter activity result in slower drug clearance and extended half-lives. As renal function improves over the first weeks of life, clearance increases, and we expect that PDAE shortens. [26] These maturational patterns are consistent with prior population PK work demonstrating lower ampicillin clearance in younger and more preterm infants. [13] Although we expect PDAE to be most pronounced in extremely premature infants during the first week of life or in those with impaired renal function, our data indicate that meaningful PDAE persists even among late preterm infants. Similar patterns may occur for other renally eliminated drugs, or possibly infants and children under certain pathophysiological states (e.g., acute or acute-on-chronic renal impairment). Simulations of several antibiotics used in EOS and late onset sepsis (LOS) in preterm infants suggest that while gentamicin (EOS) and tobramycin (LOS) are not associated with significantly prolonged PDAE, piperacillin and cefepime exposures remained therapeutic for >24 h after the final dose, on average, for MICs relevant for Enterobacterales. [14, 27] These findings identify additional opportunities for neonatal antimicrobial optimization and stewardship.

The study’s opportunistic design allowed us to capture standard-of-care drug exposures and obtain PK samples during standard-of-care lab draws in a vulnerable population, improving acceptability to families and enhancing feasibility. While well-designed prospective studies and clinical trials remain gold-standard evidence to inform neonatal antimicrobial stewardship interventions, such trials frequently exclude the most vulnerable preterm infants. For example, the NICU Antibiotics and Outcomes (NANO) trial will evaluate empiric antibiotics for EOS versus placebo in 23-30 week GA infants, but excludes high-risk infants (NCT03997266). [28] Therefore, the use of modeling and simulation followed by clinical confirmation allows for reassessment of current dosing practices to match exposures to the intended duration. With additional clinical validation and stakeholder engagement, PDAE modeling tools could be integrated into EHR and clinical decision support systems. This integration would allow real-time, patient-specific data to inform PDAE calculations. Clinicians could then view modeled exposure timelines at the point of care to inform antibiotic duration of therapy. Prior to the implementation of PDAE modeling tools at the bedside, remaining knowledge gaps should be addressed, including the clinical impact of PDAE. Additionally, engaging key stakeholders—such as clinicians, pharmacists/pharmacologists, and bioinformaticians—and addressing barriers like model validation, user training, model evaluation and improvement, [29] and cost-effectiveness will be essential for the successful adoption of PDAE modeling as a future antimicrobial stewardship tool.

There are limitations to this study. First, no infants were enrolled with GA <26 weeks, due to patient acuity and parental refusal. Because PDAE decreases with increasing GA, the summary PDAE durations and target attainments seen here are likely an underestimate of those experienced in a younger cohort (e.g., <26-week GA infants). PTA and PDAE estimates for the “Entire cohort” group are influenced by the distribution of enrolled infants and should not be viewed as absolute; had the cohort been either younger or older, these estimates would change accordingly. Second, a substantial portion of PK samples (19/49) fell outside the validated quantitative range of the assay. The three AQL samples were consistent with concentrations predicted by the population PK model and likely reflect high exposures preterm infants experience with the commonly used ampicillin dosing of 200 mg/kg/day. The high number of BQL samples is likely reflective of collection windows long after ampicillin discontinuation; these windows were informed by simulations conducted in a virtual cohort of 22 to 25 week GA infants, where PDAE is expected to be longest. [14] Though sampling volume was minimal and opportunistic, performing pre-study simulations across a broad GA range might have minimized unnecessary sample collection. An additional source of BQL values when leveraging standard of care documentation is imprecise dosing event data. We included these out-of-range values to maximize the use of all available data; however, this limited the number of available PK samples within the validated range for model evaluation. Third, ampicillin dosing at our institution was 200 mg/kg/day, and the dosing interval was not adjusted for greater degrees of prematurity. This total dose is higher than recommended dosing per the updated FDA label and AAP Red Book recommendations [30, 31], and for infants < 34 weeks GA, dosing at Q12H intervals is recommended over Q8H intervals. While 200 mg/kg/day remains a frequently used total daily dose in preterm infants, [32] dosing above the PK/PD-informed guideline-recommended total daily dose in this population, and at a more frequent interval, may contribute to longer PDAE durations. We recommend guideline-concordant dosing and expect that PDAE durations might be shorter under current FDA-approved dosing. One future aim of this work is to combine PK data from the current study and the preterm infants enrolled in the Tremoulet study to better understand PDAE durations using various dosing regimens and define the populations for which shorter empiric regimens (24- or 36-h) can be considered.

## Conclusions

Under the commonly used ampicillin dosing regimen (200 mg/kg/day administered for 48 h), ≥95% of simulated preterm infants experience therapeutic exposures for relevant Gram-positive organisms for at least 24 h after the final dose. PDAE is expected to increase with decreasing gestational age and worsening renal function and represents a period of unrecognized antibiotic exposures. Recognizing and accounting for PDAE represents a novel opportunity for neonatal antimicrobial stewardship. Future work should identify populations with prolonged PDAE under FDA-recommended dosing and prospectively evaluate the PK and safety of short-course (24-h) empiric ampicillin.

## Supplementary information

Supplementary materials

## Data Availability

The datasets in this study can be made available upon request.
