# Supplementary material for: Evaluating the duration of post-discontinuation therapeutic ampicillin exposures in preterm infants
Source: J Perinatol. 2026 Mar 13;46(6):1051–8. doi: 10.1038/s41372-026-02600-5 (PMC13290478; doi:10.1038/s41372-026-02600-5)
Supplement: Supplementary file 1 — Supplementary materials [file 41372_2026_2600_MOESM1_ESM.docx]

**Supplementary materials**

Table of Contents

[**Supplemental Figure S1:** Observed and predicted ampicillin concentrations over time, by subject 2](#_Toc205416058)

[**Supplemental Table S1**: Demographic comparisons across three ampicillin pharmacokinetic studies 4](#_Toc205416059)

##
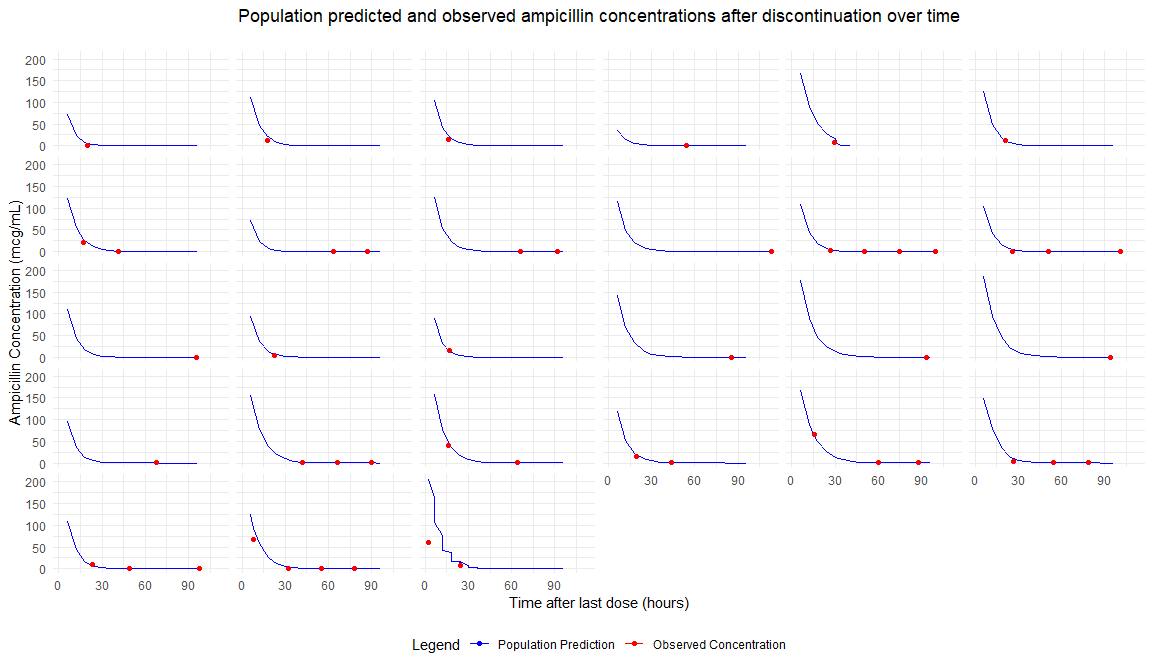
**Supplemental Figure S1:** Observed and predicted ampicillin concentrations over time, by subject

**Figure legend:** Each graph displays the post-discontinuation ampicillin concentrations (observed concentrations in red, population predicted concentrations in blue) over time, for each individual infant. Observed concentrations (N=49) were collected from 27 infants after the final dose of ampicillin and underwent high-performance liquid chromatography-mass spectrometry for quantitation of ampicillin concentrations. The validated assay range was 0.5 to 50 μg/mL. Population predicted concentrations were simulated using the published Tremoulet, et al., model to predict exposures every 6 hours from 6 to 96 hours after the final dose of ampicillin using NONMEM 7.3 (Icon, Dublin, Ireland).

## **Supplemental Table S1**: Demographic comparisons across three ampicillin pharmacokinetic studies

|  | **Tremoulet, et al^1^** | **Le et al^2^** | **Current study** |
| --- | --- | --- | --- |
| N | 48 (<7d PNA)  25 (≥7d PNA) | 34,689 virtual infants | 27 |
| GA, median | 32.3 (<34 week)  38 (≥34 week) | 26 | 31w3d |
| Min GA, Max GA | 24, 34  34, 41 | 22, 27 | 26w6d, 36w6d |
| BW | Not reported | 790 (400-1497) | 1730 (870, 2940) |
| PNA, median [Q1, Q3] | 1 (0, 7) | 1 | 0 [0,1] |
| PDAE estimates for MIC target of 1 μg/mL | NA | Dosing: 200mg/kg/d divided Q12H, 4 total doses  Mean (95% confidence interval [CI]) PDAE:  74 hours (95% CI 35, 109) | Dosing: 200mg/kg/d divided Q8H, 6 total doses  Median (95% CI) PDAE:  < 28 weeks – 53.2 (26.1, 101)  28 to < 34 wk GA – 37.5 (17.2, 74.7)  34 – 36 wk GA – 26.6 (11.4, 53.7) |
| BW – birth weight, GA – gestational age, MIC – minimum inhibitory concentration, PDAE – post-discontinuation antibiotic exposures, PNA – postnatal age  ^1^ Tremoulet A, Le J, Poindexter B, et al. Characterization of the Population Pharmacokinetics of Ampicillin in Neonates Using an Opportunistic Study Design. *Antimicrobial Agents and Chemotherapy*. 2014;58(6):3013-3020. doi:doi:10.1128/aac.02374-13  ^2^ Le J, Greenberg RG, Benjamin DK, et al. Prolonged Post-Discontinuation Antibiotic Exposure in Very Low Birth Weight Neonates at Risk for Early-Onset Sepsis. *Journal of the Pediatric Infectious Diseases Society*. 2021;10(5):615-621. doi:10.1093/jpids/piaa172 | | | |
